# Cytokinins Are Abundant and Widespread among Insect Species

**DOI:** 10.3390/plants9020208

**Published:** 2020-02-06

**Authors:** Peter Andreas, Anna Kisiala, R. J. Neil Emery, Rosemarie De Clerck-Floate, John F. Tooker, Peter W. Price, Donald G. Miller III, Ming-Shun Chen, Edward F. Connor

**Affiliations:** 1 Department of Biology, Trent University, Peterborough, ON K9J 7B8, Canada; pandreas@trentu.ca (P.A.); annakisiala@trentu.ca (A.K.); nemery@trentu.ca (R.J.N.E.); 2 Agriculture and Agri-Food Canada, Lethbridge, AB T1J 4B1, Canada; rosemarie.declerck-floate@canada.ca; 3 Department of Entomology, The Pennsylvania State University, University Park, PA 16802, USA; tooker@psu.edu; 4 Department of Ecology and Evolutionary Biology, Northern Arizona University, Flagstaff, AZ 86001, USA; Peter.Price@nau.edu; 5 Department of Biological Sciences, California State University, Chico, CA 95929, USA; DGMiller@csuchico.edu; 6 USDA-ARS and Department of Entomology, Kansas State University, Manhattan, KS 66506, USA; ming-shun.chen@usda.gov; 7 Department of Biology, San Francisco State University, San Francisco, CA 94132, USA

**Keywords:** cytokinins, gall-inducing, non-gall inducing, insects, phytophagous

## Abstract

Cytokinins (CKs) are a class of compounds that have long been thought to be exclusively plant growth regulators. Interestingly, some species of phytopathogenic bacteria and fungi have been shown to, and gall-inducing insects have been hypothesized to, produce CKs and use them to manipulate their host plants. We used high performance liquid chromatography-electrospray ionization tandem mass spectrometry (HPLC-MS/MS) to examine concentrations of a wide range of CKs in 17 species of phytophagous insects, including gall- and non-gall-inducing species from all six orders of Insecta that contain species known to induce galls: Thysanoptera, Hemiptera, Lepidoptera, Coleoptera, Diptera, and Hymenoptera. We found CKs in all six orders of insects, and they were not associated exclusively with gall-inducing species. We detected 24 different CK analytes, varying in their chemical structure and biological activity. Isoprenoid precursor nucleotide and riboside forms of trans-zeatin (*t*Z) and isopentenyladenine (iP) were most abundant and widespread across the surveyed insect species. Notably, the observed concentrations of CKs often markedly exceeded those reported in plants suggesting that insects are synthesizing CKs rather than obtaining them from the host plant via tissue consumption, compound sequestration, and bioaccumulation. These findings support insect-derived CKs as means for gall-inducing insects to manipulate their host plant to facilitate cell proliferation, and for both gall- and non-gall-inducing insects to modify nutrient flux and plant defenses during herbivory. Furthermore, wide distribution of CKs across phytophagous insects, including non-gall-inducing species, suggests that insect-borne CKs could be involved in manipulation of source-sink mechanisms of nutrient allocation to sustain the feeding site and altering plant defensive responses, rather than solely gall induction. Given the absence of any evidence for genes in the de novo CK biosynthesis pathway in insects, we postulate that the *t*RNA-*ipt* pathway is responsible for CK production. However, the unusually high concentrations of CKs in insects, and the tendency toward dominance of their CK profiles by *t*Z and iP suggest that the *t*RNA-*ipt* pathway functions differently and substantially more efficiently in insects than in plants.

## 1. Introduction

Cytokinins (CKs) are members of a class of signaling molecules often referred to as plant growth regulators or phytohormones. In plants, CKs regulate cell differentiation, cell division, apical dominance, delay of senescence, sink strength, and a variety of other processes [1,2,3]. CKs are not exclusively plant-derived compounds. CKs have been reported from a variety of organisms including bacteria [4,5,6,7,8,9], fungi [10,11,12], protists [13], nematodes [14], insects [15,16,17,18,19], and mammals [20], including humans [21]. CKs can be seen as inter-kingdom signaling molecules that orchestrate cross-talk with plants but may also display bioactive functions not related to interactions with plants. Beyond plants, CK biosynthesis pathways have been described in bacteria [6,8,10,22,23,24,25,26] and fungi [11,27,28]. In other taxa, including insects, however, the origin of these compounds remains unclear.

Adenine-derived CKs such as trans-zeatin (*t*Z), cis-zeatin (*c*Z), dihydrozeatin (DHZ), and isopentenyladenine (iP) can be found in the forms of active free bases (FBs), and their various conjugates: ribosides (RBs), nucleotides (NTs), methyl-thiols (METs), and glucosides (GLUCs). There are two known pathways for the biosynthesis of isoprenoid CKs, and knowledge of their structure and function derives almost entirely from studies in plants. First, the *t*RNA-*ipt* pathway is ubiquitous to all organisms except the Archaea [29,30,31]. The *t*RNA-*ipt* gene (*t*RNA dimethylallyltransferase; EC: 2.5.1.75) modifies an adenine residue at position 37 of a *t*RNA by adding an isoprenoid chain to increase the fidelity of translation [32,33]. In plant plastids and bacteria, the methyl erythritol (MEP) pathway is the main source of the prenyl group, but in the cytosol of plants and in insects, the prenyl group is synthesized via the mevalonate pathway (MVA) [34,35]. Upon degradation of the prenylated *t*RNA, CK is produced. In plants, the *t*RNA-*ipt* pathway has long been thought to produce exclusively *c*Z type CK [36]. However, recent work with the gall-inducing fungus *Ustilago maydis* and other fungi, along with the pathogenic bacterium *Mycobacterium tuberculosis,* suggest that in addition to *c*Z, both isopentenyladenine (iP) and methylthiolated CKs (METs) can also be produced via the *t*RNA-*ipt* pathway [6,11,27].

Apart from the *t*RNA-*ipt* pathway, plants, gall-inducing bacteria, and some fungi also possess the de novo biosynthesis pathway. In the de novo pathway, the gene adenylate dimethylallyltransferase (EC: 2.5.1.27 and EC: 2.5.1.112) leads to the production of iP, *t*Z, and DHZ-type CKs [10,36,37]. In plants and in the pathogenic fungus *Claviceps purpurea*, cytochrome P450 monooxygenases convert iP to *t*Z [10,38]. *t*Z can be further modified to DHZ by zeatin reductase enzymes [2]. The first products of both CK pathways are the inactive NT forms. NTs are subsequently activated directly to FBs by the *log* gene (cytokinin riboside 5′-monophosphate phosphoribohydrolase, EC: 3.2.2) or indirectly, through the RB stage, by genes with phosphatase or phosphoribohydrolase activity [6,8,10,29].

In organisms that have been found to contain CKs and appear to manipulate plants, exogenous CKs have been implicated as contributing to several effects on the host plant. The most apparent of these effects is that CKs in the presence of auxin lead to cell division and proliferation of plant tissue resulting in the formation of galls or tumors by gall-inducing taxa. To date, the only definitive evidence of a role for CKs in the proliferation of gall tissue in plants exists for bacteria [4,25,39,40,41]. However, CKs have long been implicated as contributing to the formation of the covering gall in fungi [11,12,42,43] and insects [15,16,19,44,45,46,47,48,49,50]. By covering gall, we mean the plant tissue that envelopes the gall-inducing organism. Among fungi and insects that encounter senescing plant tissues, some species appear to employ CKs to induce yellowing plant tissues to “stay green” by the process of forming “green islands”—areas of photosynthetically active tissues in which the pathogenic organism can continue to feed and develop [17,51,52,53,54,55,56,57]. In some instances, CKs have been shown to convert the site of attack into a mobilizing sink to which sugars are translocated where they may contribute to gall induction or just for the sustenance of the manipulating organism [18,28,45,58,59,60].

In addition to altering flow of nutrients, CKs can also influence plant defensive responses [61,62]. With herbivory, levels of CKs can increase and there is a corresponding ramping up of CK-regulated genes that are involved in plant defensive responses against attacking insects, including suppression of some indirect defenses [62]. Moreover, there is good evidence that a range of plant antagonists, such as bacteria, viruses, fungi, and insects—mainly leaf miners and gall inducers—produce CKs to facilitate invading the plant, perhaps in part by counteracting plant defensive responses [61]. Finally, exogenous CKs, in the presence of light, appear to modify biosynthesis of flavonoids and anthocyanins via altered regulation of the phenylpropanoid pathway, resulting in the synthesis of red and purple pigments that sometimes color the infection site or gall and that may also increase the production of tannins and lignins [45,63,64,65].

The earliest studies used bioassays to confirm the presence of CKs in insects [66,67,68,69,70]. Since then, researchers have used either ELISA [52,54,55], or analytical chemistry [15,16,17,19,44,46,47,49,50,57], to estimate the concentrations of CKs in insects.

Detection of CKs in insects has been almost exclusively associated with two phenotypic alterations in the host plant: production of so called “green islands” by leaf-mining Lepidoptera, and production of galls or tumors by gall-inducing insects. Although the literature lacks reports of CKs in insect species within other phytophagous or non-phytophagous feeding guilds (except for [18]), this could represent a lack of exploration rather than the absence of CKs outside the leaf-mining Lepidoptera and gall-inducing Diptera, Hymenoptera, and Hemiptera.

We examined the CK profiles of a wide variety of phytophagous insects from all six orders that contain species known to induce galls: Thysanoptera, Hemiptera, Lepidoptera, Coleoptera, Diptera, and Hymenoptera. Of these six orders, four orders are also known to have evolved the ability to mine leaves: Lepidoptera, Coleoptera, Diptera, and Hymenoptera [71]. We selected species that are known to induce galls, and closely related insect species from the same orders that do not induce galls. Our goal was to determine if high concentrations of CKs were always associated with gall induction, or if CKs also occur in non-gall-inducing insects.

## 2. Materials and Methods

### 2.1. Insect Species

We collected 17 species of gall-inducing and closely related non-gall-inducing species from six orders of Insecta for CK profiling. We analyzed the gall-inducing stage, or among non-gall-inducing species, the analogous stage, while also examining other life stages for a few species (Table 1). The gall-inducing stage is often the early instar larvae, which can be very small (<0.2 mg). Therefore, large numbers are required to obtain enough tissue for phytohormone analyses, i.e., >50 mg of fresh weight in total to achieve replication (at least three biological replicates). For some insects, we report the CK levels for both the juvenile and adult female stages, depending on their life cycle and their involvement with the host plant.

Insect species were selected based on their availability in numbers sufficient for CK analyses, so that pairs of gall- and non-gall-inducing species were as closely related as possible. Most species pairs were from the same taxonomic family; however, in one case, a congeneric pair of gall- and non-gall-inducing species was selected (*Tamalia* spp.). Insects were either wild-collected or reared, stored at −80 °C, and shipped on dry ice for analysis.

### 2.2. Cytokinin Analysis by High Performance Liquid Chromatography-Electrospray Ionization Tandem Mass Spectrometry (HPLC-(ESI)-MS/MS)

Chemical analysis was conducted for 29 forms of cytokinins (CKs) in insects (Table 2). CKs were extracted and quantified using methods slightly modified from those described in [72,73]. No procedure was practicably possible to clear the insects’ gut before analysis. Insect samples were spiked with 10 ng of deuterated internal standards (OlChemim Ltd., Olomouc, Czech Republic) and homogenized in 1.0 mL of −20 °C modified Bieleski #2 extraction solvent (methanol: water: formic acid (15:4:1, v/v/v)) using sterile zirconium oxide grinding beads (Comeau Technique Ltd., Vaudreuil-Dorion, Canada) and a Retsch 400 mixer-mill (Retsch, Haan, Germany) at 25 Hz for 5 min. Samples were passively extracted at -20 °C for 12 h, then centrifuged for 10 min at 10,000 RPM (Thermo Scientific; Model Sorvall ST16, Ottawa, Canada) and the supernatant was collected. The remaining pellet was re-extracted using 1 mL of modified Bieleski #2 solvent at −20 °C for 1 h and the two supernatants were combined. Supernatants were dried down in a speed vac concentrator at 35 °C (Savant SPD111V, UVS400, Thermo Fisher Scientific, Waltham, MA). Residues were reconstituted in 0.2 mL of 1M formic acid (pH 1.4) to ensure complete protonation of all CKs. Extracts were purified and fractionated on a mixed mode, reverse-phase, cation-exchange cartridge (Waters; Oasis MCX 2cc; 96-well plate, Mississauga, ON, Canada) using an automated liquid handler system (Gilson Liquid Handler, Model 215 SPE System, Middleton, WI, USA). Cartridges were activated using 1 mL of HPLC grade methanol followed by equilibration using 1 mL of 1M formic acid (pH 1.4). Reconstituted samples were loaded into the cartridges after equilibration. Bound residues were washed with 1 mL of 1M formic acid (pH 1.4) and 1 mL of methanol. The nucleotide (NT) fraction of CKs was eluted first using 1 mL of 0.35M ammonium hydroxide. Free bases (FBs), ribosides (RBs), methyl-thiols (METs), and glucosides (GLUCs) were eluted last using 1 mL of 0.35 M ammonium hydroxide in 60% methanol. The extracts were dried down in a speed vac concentrator at 35 °C and stored at −20 °C until further processing.

The NT fraction was dephosphorylated prior to HPLC-MS/MS analysis using 3 units of alkaline phosphatase (New England BioLabs, alkaline phosphatase calf intestine, Whitby, Ontario, Canada) in 1 mL of 0.1 M ethanolamine-HCL (pH 10.4) for 12 h at 37 °C. The resulting RBs were brought to dryness in a speed vac concentrator at 35 °C. The residues were re-constituted in 0.3 mL double distilled Milli-Q water for further purification on C18 cartridges (Canadian Life Sciences; C18, 2cc, 96-well plate; Peterborough, ON, Canada) that were activated with 1 mL HPLC grade methanol and equilibrated with 1 mL double distilled Milli-Q water prior to loading the samples. Bound residues were washed with 1 mL double distilled Milli-Q water and eluted using 1 mL HPLC grade methanol. All sample eluents were dried down in a speed vac concentrator. All dried CK fractions were re-constituted in 1.5 mL of the starting conditions solution (Milli-Q water: acetonitrile: acetic acid; 94.92:5:0.08, v/v/v).

CKs were analyzed by HPLC-(ESI)-MS/MS on a QTrap5500 triple quadrupole mass spectrometer (Sciex Applied Biosystems, Massachusetts, United States) connected to an Agilent 1100 series HPLC. Detection limits are as given in [73]. These methods have been previously applied to profile CKs in plant galls and gall-inducing insects [16]. The levels of 29 CKs are reported as the average of 3 to 6 replicates ± standard error, in picomoles per gram fresh weight of the insect tissue (pmol/g fwt). We performed analyses on whole insects or groups of insects, so the reported CK levels reflect concentrations averaged across all body tissues.

## 3. Results and Discussion

### 3.1. Cytokinin Profiles and Levels in Insects

**Figure 1:**
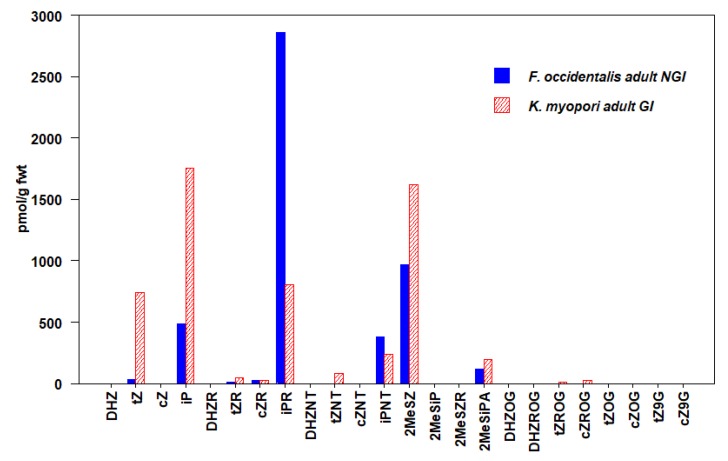
Concentrations of cytokinins (CKs) in Thysanoptera (thrips). Values are means expressed as pmol/g fwt of whole-body mass in gall-inducing *K. myopori* (hatched bars) and non-gall-inducing *F. occidentalis* (solid bars) (Means ± standard errors are given in Table S1).

**Figure 2:**
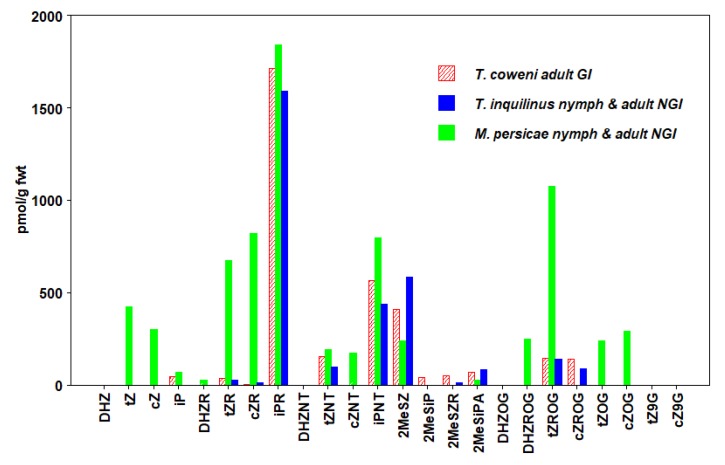
Concentrations of cytokinins (CKs) in Hemiptera (aphids). Values are means expressed as pmol/g fwt of whole-body mass in gall-inducing *T. coweni* (hatched bars) and non-gall-inducing *T. inquilinus* and *M. persicae* (solid bars) (Means ± standard errors are given in Table S1).

**Figure 3:**
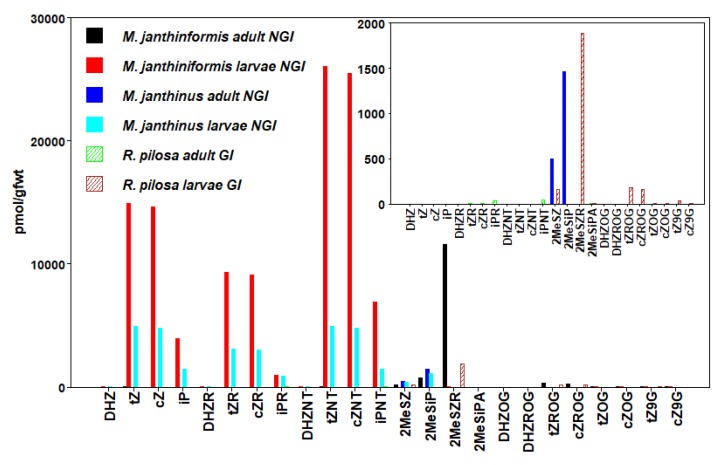
Concentrations of cytokinins (CKs) in Coleoptera (beetles). Values are means expressed as pmol/g fwt of whole-body mass in gall-inducing *R. pilosa* (hatched bars) and non-gall-inducing *M. janthiniformis* and *M. janthinus* (solid bars). Bar graphs in upper right corner show concentrations of CKs for species with relatively low concentrations that are not readable on larger plot (Means ± standard errors are given in Table S2).

**Figure 4:**
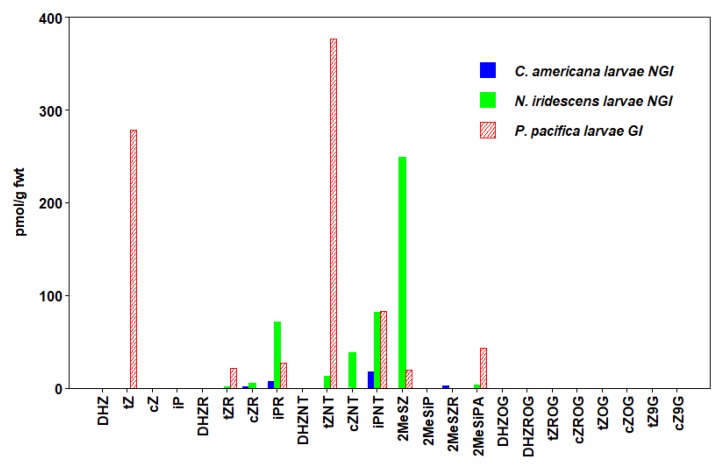
Concentrations of cytokinins (CKs) in Hymenoptera (sawflies). Values are means expressed as pmol/g fwt of whole-body mass in gall-inducing *P. pacifica* (hatched bars) and non-gall-inducing *N. iridescens* and *C. americana* (solid bars) (Means ± standard errors are given in Table S3).

**Figure 5:**
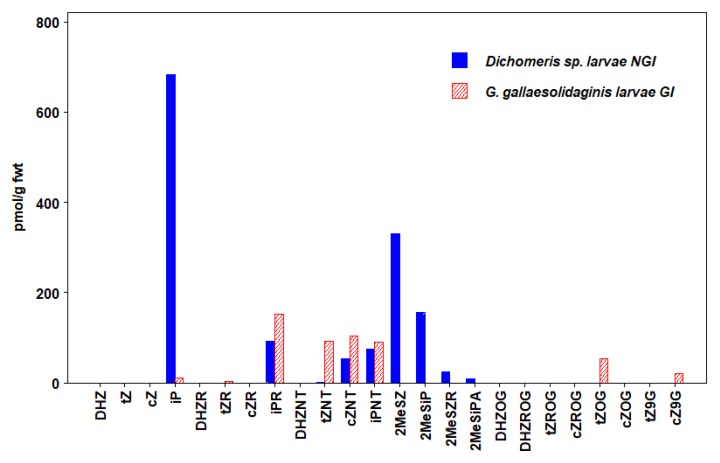
Concentrations of cytokinins (CKs) in Lepidoptera (moths). Values are means expressed as pmol/g fwt of whole-body mass in gall-inducing *G. gallaesolidaginis* (hatched bars) and non-gall-inducing *Dichomeris* sp. (solid bars) (Means ± standard errors are given in Table S3).

**Figure 6:**
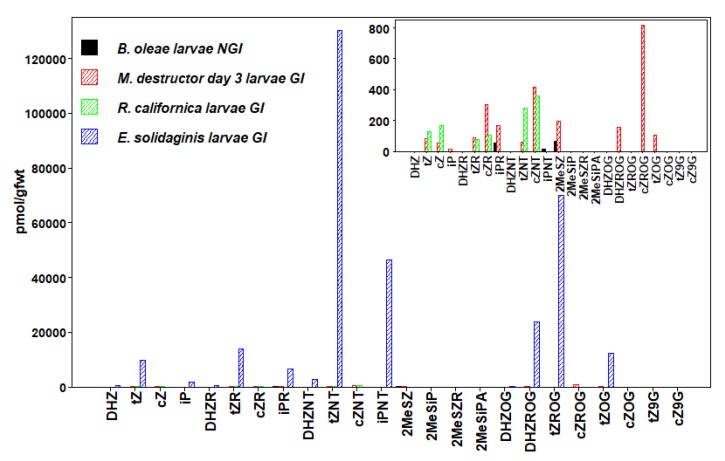
Concentrations of cytokinins (CKs) in Diptera (flies). Values are means expressed as pmol/g fwt of whole-body mass in gall-inducing *E. solidaginis*, *M. destructor*, and *R. californica* (hatched bars) and non-gall-inducing *B. oleae* (solid bars). Bar graphs in upper left corner show concentrations of CKs for species with relatively low concentrations that are not readable on larger plot (Means ± standard errors are given in Table S4).

We detected 24 types of CKs in insects, including free bases (FBs), ribosides (RBs), and nucleotides (NTs), as well as methyl-thiols (METs) and glucosides (GLUCs) (Figure 1, Figure 2, Figure 3, Figure 4, Figure 5 and Figure 6, Table 3, Supplementary Tables S1–S4). CKs were detected in all six orders of insects and were not exclusively associated with gall-inducing species. RBs and NTs were most abundant and widespread. In most species, FB, RB, and NT forms of *t*Z and iP dominated the CK profiles. FB forms of CKs were found in both gall-inducing and non-gall-inducing insects, although they were more common in gall-inducing species. Glucosides, considered as CK storage forms, were generally uncommon (Supplementary Tables S1–S4). CK forms for which we performed analyses, but that were not detected in any species of insect include dihydro-zeatin-9-glucoside (DHZ9G), isopentenyladenine-7-glucoside (iP7G), isopentenyladenine-9-glucoside (iP9G), benzylaminopurine (BA), and benzylaminopurine riboside (BAR).

### 3.2. Thysanoptera

The Thysanoptera is the only order for which we were unable to collect the gall- and non-gall-inducing species from the same family (Table 1). The gall-inducing *Klambothrips myopori* Mound & Morris, in which the adults induce rapid leaf distortion resulting in leaf-fold galls and stunted growth on *Myoporum laetum* G. Forst. shrubs (Scrophulariaceae) [74], belongs to the Phlaeothripidae, and its non-gall-inducing comparison species, the western flower thrips, *Frankliniella occidentalis* (Perg.) is from the Thripidae [75]. We detected 11 different types of CKs within the gall-inducing adults of *K. myopori*, and 8 of these were also found in adult *F. occidentalis* at similar concentrations (Figure 1, Table S1). Out of tZ-, cZ-, and iP-type CKs, in both species, iP forms were most abundant, and they were at higher levels in the non-gall-inducing *F. occidentalis*. Interestingly, gall-inducing *K. myopori* contained notably higher levels of *t*Z forms in comparison, contributing to the higher total CK concentration in this species (Table 3). The highest levels of 2-methylthio-zeatin (2MeSZ) were detected in *K. myopori,* and, secondarily, in *F. occidentalis* compared to any of the other species evaluated.

### 3.3. Hemiptera

In the Hemiptera, we examined two species of aphids that feed on shrubs in the genus *Arctostaphylos* (Ericaceae) [76,77,78]. In one of these species, *Tamalia coweni* (Cockerell) (Aphididae), the stem mother induces leaf-roll galls on the margins of young leaves. Within this gall, the nymphs are produced and feed until maturity. In late-stage galls, one can also find nymphs and adults of the sister species, *Tamalia inquilinus* (Miller), which as an inquiline, occupies galls induced by *T. coweni* to share the food resource, but does not induce galls [77]. *Tamalia inquilinus* is derived from the clade of gall-inducing *T. coweni* [79,80]. We also examined adults and nymphs of the green peach aphid, *Myzus persicae* (Sulzer), which feed on a wide variety of host plants and do not induce galls (Table 1).

We detected a wide range of CKs in all three species of Hemiptera. The highest number of CK types and the highest total CK concentrations were detected in a mixture of adults and nymphs of the non-gall-inducing *M. persicae* (Figure 2, Table S1). The total CK levels in *M. persicae* were higher than in a mixture of adults and nymphs of *T. inquilinus* and stem mothers of *T. coweni* by 2- and 3-fold, respectively (Table 3). Among all three species, iP forms were most abundant, but several forms of *t*Z and *c*Z were also found in all species (Figure 2, Table 3). The less common DHZ forms including dihydrozeatin riboside (DHZR) and its storage form dihydrozeatin-O-glucoside riboside (DHZROG) were only detected in *M. persicae* (Figure 2). The CK profiles of the two species of *Tamali*a were strikingly similar. Both species presented CK profiles containing 10 common CK types in comparable concentrations, excluding iP and 2-methylthio-isopentyladenine (2MeSiP), which were only found in the gall-inducing *T. coweni.* This suggests that the CK production by *T. coweni* is not the sole condition for gall induction; however, we cannot exclude the option that iP and 2MeSiP are critical for that purpose.

Our results are similar to values reported by [16] who assayed for a wide spectrum of CKs in the nymphs of gall-inducing *Pachypsylla celtidismamma* Riley (Hemiptera: Psyllidae). However, the CK concentrations in *P. celtidismamma* exceeded those that we report by approximately 30%. Fundatrices of the gall-inducing *Tetraneura nigriabdominalis* Sasaki (Hemiptera: Aphididae) and nymphs of the gall-inducing *Stenopsylla nigricornis* Kuwayama (Hemiptera: Triozidae) were examined for iP, iPR, *t*Z, and *t*ZR [49,50]. All these CKs were detected, but at levels lower than we found in *Tamalia* or *Myzus*. Another study assayed for 6 forms of CKs in adults of the free-living phytophagous bug *Tupiocoris notatus* Distant (Hemiptera: Miridae) and reported summed concentrations ranging between 1 and 16 pmol/g fwt, with iP being the most abundant [18].

### 3.4. Coleoptera

In the Coleoptera, we examined larval and adult stages of three species of weevils (Curculionidae) within the tribe Mecinini: the stem-galling *Rhinusa pilosa* (Gyllenhal), and stem-boring *Mecinus janthinus* Gemar and *Mecinus janthiniformis* Tosevski & Caldera, all closely affiliated with host plant species in the genus *Linaria* (Plantaginaceae) [81,82]. Female *R. pilosa* induce their galls via an oviposition fluid deposited with their eggs into cavities that they pre-excavate within the growing stems of yellow toadflax, *Linaria vulgaris* Miller, and the resultant large, fleshy galls are fully formed before the eggs hatch [83]. In the case of the stem-boring species, *M. janthinus* and *M. janthiniformis*, females similarly excavate cavities in stems prior to laying their eggs within their hosts, in this study, yellow toadflax and Dalmatian toadflax (*Linaria dalmatica* (L.) Miller), respectively (Table 1). After hatching, the larvae produce tunnels within the stems as they feed.

In *R. pilosa*, where the gall is entirely induced by the adult female, larvae had no active FBs or precursor RBs and NTs of any type, displaying entirely METs and the storage form GLUCs. Nine types of CKs were found in the larvae of this species, with a MET form of ZR being the most abundant (Figure 3, Table S2). Adult *R. pilosa* females were collected during peak oviposition. Five types of CKs were detected in low abundance in the adult females of *R. pilosa* with the clear dominance of iP types. This trend to some extent resembles the CK profile of adult females of *E. solidaginis* where iPR was the most abundant CK form detected.

The larvae of the stem-boring weevil *M. janthiniformis* had the second highest concentrations of total CKs detected, after the gall-inducing fly *E. solidaginis*. *Mecinus janthiniformis* larvae had very high concentrations of NT, RB, and FB forms of both *t*Z and *c*Z, with lower concentrations of iP (Figure 3, Table S2). The adult females of *M. janthinus* and *M. janthiniformis* had low levels of FBs, RBs, and NTs, and higher levels of METs and GLUCs (Table 3). Compared to all the other insects we surveyed, adults of *Mecinus* species had the highest total level of METs.

Regarding FB, RB, and NT forms, the larvae of non-gall-inducing *M. janthinus* had a CK profile similar to *M. janthiniformis* larvae, but at concentrations 2–5 fold lower than *M. janthiniformis*. Although the weevil species are very similar morphologically and genetically, and are both defined as stem-borers based on their general larval feeding habit, the two can be distinguished by some subtle biological characteristics [82]. *Mecinus janthiniformis* oviposits into younger host stem tissues and produces a slight “gall-like” swelling external to where its larvae feed internally within stems of its hosts, whereas *M. janthinus* oviposits into older stem tissues and produces no such swellings. It also was noted by [82] that the larval feeding tunnels within infested host stems were generally shorter for *M. janthiniformis* than for *M. janthinus*, thus suggesting that the larvae of the former species are more efficient in manipulating the source-sink mechanism of its host in drawing nutrients to themselves, perhaps with the use of elevated CKs.

### 3.5. Hymenoptera

In the Hymenoptera, we examined the profiles of CKs in larvae of three species of sawflies (in the superfamily Tenthredinoidea). The galls of *Pontania pacifica* Marlatt are initially induced in young developing leaves of arroyo willow, *Salix lasiolepis* Benth. (Salicaceae), by the adult female at oviposition, but further growth of the gall occurs during feeding by the larvae [66,84]. We also examined larvae of the non-gall-inducing *Nematus iridescens* Cresson, which is from the same subfamily as *Pontania* (Tenthredinidae: Nematinae) and feeds on the leaves of *Populus* L. (Salicaceae), and larvae of a leaf-chewing sawfly from a different family, *Cimbex americana* Leach (Tenthredinoidea: Cimbicidae), which feeds on *S. lasiolepis* (Table 1).

Of the 7 CK types present in the profile of *P. pacifica,* 6 were also found in the subfamilial relative, *N. iridescens* (Figure 4, Table S3). Total CKs and *t*Z forms were the most abundant in the gall-inducing *P. pacifica*, while the iP forms made up most of the FB, RB, and NT pool in the two non-gall-inducing sawfly species (Table 3). The non-gall-inducing *C. americana* showed a CK profile of only 4 CK types and contained the lowest total CKs of all the insects surveyed.

The total CK concentration for the larvae of a *Pontania* sp. previously examined by [46] was 6-fold higher than for *P. pacifica* in this study, yet the profiles were similar with respect to the level of *t*Z forms relative to other types of CKs. However, the concentrations of CKs detected in this study for *P. pacifica*, were 4-fold higher than the concentrations reported by [44] for larvae of the gall-inducing *Trichilogaster acaciaelongifoliae* Froggatt (Hymenoptera: Pteromalidae), assuming the larval body moisture content is 61% [85].

### 3.6. Lepidoptera

Analysis of larvae of two moth species that feed on *Solidago altissima* L. (Asteraceae), the stem-galling *Gnorimoschema gallaesolidaginis* (Riley), and the leaf-chewing *Dichomeris* sp. Hübner (Table 1) revealed the presence of nine types of CKs in *Dichomeris* sp., five of which were also common in *G. gallaesolidaginis*. Forms of *t*Z, *c*Z, and iP were detected in both caterpillar species (Figure 5, Table S3). Forms of iP were the most abundant CKs in both species, but the non-gall-inducing *Dichomeris* sp. had a 4-fold higher concentration of iP forms, and 3-fold higher concentrations of *t*Z forms than the gall-inducing *G. gallaesolidaginis*, while *c*Z forms were found in higher levels within *G. gallaesolidaginis*. The pool of iP and *t*Z forms contributed to the higher level of total CKs detected in *Dichomeris* sp. (Table 3). All 4 MET forms were detected in *Dichomeris* sp. Besides *Dichomeris* sp., the gall-inducing aphid, *T. coweni*, was the only other species we examined that contained all 4 MET forms of CK.

In the green-island inducing and leaf-mining larvae of *Phyllonorycter blancardella* Fabricius (Lepidoptera: Gracillariidae), [17] reported concentrations for a small subset of the CKs we examined. Using an estimated body moisture content of 80% [85,86], the data conversion suggests that *P. blancardella* had total CK concentrations that were approximately an order of magnitude higher than in the gall-inducing *G. gallaesolidaginis* and also higher than the free-living *Dichomeris* sp. Furthermore, in *P. blancardella t*Z, *t*ZR, and iP were the most abundant CKs.

### 3.7. Diptera

Within the Diptera, we examined four species of insects, two from each of the families: Tephritidae and Cecidomyiidae. In the Tephritidae, we examined the gall-inducing *Eurosta solidaginis* (Fitch) and the non-gall-inducing olive fly, *Bactrocera oleae* Rossi. Ovipositing *E. solidaginis* insert the egg into the apical meristem of *S. altissima* (Asteraceae). Upon hatching, the larvae induce the formation of a ball-shaped gall in the stem of the host-plant. The olive fly does not induce a gall, but oviposition occurs into a developing fruit and the larvae feeds within. In the Cecidomyiidae, we examined the gall-inducing Hessian fly, *Mayetiola destructor (*Say), which does not form a covering gall as do most other gall-inducing insects, but it induces nutritive cell formation at the feeding site and inhibits plant growth. The Hessian fly mainly feeds on wheat, *Triticum aestivum* L. (Poaceae), and after the 2nd day of feeding, the larvae become sessile feeding between the leaf sheath and stem [87]. We also examined the gall-inducing *Rhopalomyia californica* Felt whose larvae induce communal galls (with as many as 100 larvae) on buds of coyote bush, *Baccharis pilularis* DC. (Asteraceae), forming a substantial covering gall [88] (Table 1).

In the Tephritidae, we detected 12 types of CKs in larvae of the gall-inducing *E. solidaginis*, with concentrations of NT forms of iP and *t*Z that were between 30 and 100-fold higher than the remaining 10 forms of CKs. The adult female profile revealed only 5 CKs, which included NT and RB forms of iP and *t*Z as well as their two MET conjugates. *E. solidaginis* larvae also had substantial amounts of *t*Z and DHZ glucosides. Total CK levels detected in *E. solidaginis* larvae were the highest of all the insect samples surveyed in our study (Figure 6, Table 3, Table S4). The values reported for the forms of *t*Z and iP in *E. solidaginis* are much higher than those reported by [15]. We suspect this is largely the result of differences in the extraction and analytical procedures, but could also reflect differences in CK levels between the populations sampled by [15] in New York and the populations we sampled in Minnesota. Gall-inducing *E. solidaginis* larvae contained significantly higher CK levels than its adult form. However, the CK profiles of the adult female, although low in concentration, were dominated by *t*Z and iP, as well as the MET forms, similar to its larvae. In the non-gall-inducing *B. oleae,* both larvae and adults had very low concentrations of only 3 forms of CKs (Table S4).

In the Cecidomyiidae, the level of total CKs in Hessian fly larvae was low, which is consistent with the absence of a covering gall. However, when comparing the 12 types of CKs detected, we observed that the concentrations of active FBs and RB precursors of *t*Z, *c*Z, and iP were the highest in day 1 larvae and all decreased substantially in day 3 larvae. On the other hand, levels of the precursor NTs showed a slight increase, while the levels of storage GLUCs were markedly increased from day 1 to day 3 (Table S4). The higher total CK level in the day 1 larvae comprised a suite of active CKs possibly geared toward nutritive layer induction, while the day 3 CK profile revealed that the active forms had been depleted and that larval CK production shifted toward storage conjugates. For larvae of the gall-inducing *R. californica*, the CK profile consisted of low levels of 6 forms of CKs including NT, RB, and FB forms of *t*Z and *c*Z (Figure 6, Table S4).

### 3.8. Cytokinins in Insects

Clearly CKs are not an exclusively plant-based class of compounds. In fact, the results reported here combined with earlier work on insects [15,16,17,18,44,46,47,49,50] suggest that CKs are abundant and widespread among phytophagous insect species. We report the highest whole-body concentrations of total CKs found in insects, 318,405 and 111,630 pmol/g fwt, for larvae of *E. solidaginis* and *M. janthiniformis*, respectively. In a survey of 139 species of plants, the mean total CK concentration was 81 pmol/g fwt and the median concentration was only 30.5 pmol/g fwt [89]. The average concentrations of total CKs in all the species of gall- and non-gall-inducing insects that we examined, except the sawfly *C. americana*, exceeded the average concentrations of CKs in plants. However, even the value for *C. americana* was on par with the median concentration of CKs observed in plants.

### 3.9. Evidence for Cytokinin Biosynthesis Pathway in Insects

Given the often very high concentrations of CKs in insects, it is most likely that insects are producing these compounds rather than acquiring them via consumption and sequestration from plant tissues. Currently, the NCBI Protein database includes 290 entries from the search—‘*t*RNA dimethylallyltransferase’ and ‘Insecta’ (January 27, 2020), but no entries for ‘adenylate dimethylallyltransferase’ and ‘Insecta’. However, this database does include some duplicate entries and some bacterial and fungal contamination, so the number of unique entries for Insecta is less than the total. Furthermore, the KEGG ontology database, which is based only on completely sequenced genomes, lists 65 different versions of the *t*RNA dimethylallyltransferase gene (EC: 2.5.1.75) in the Insecta, and none for adenylate dimethylallyltransferase (2.5.1.27 and 2.5.1.112) [90,91,92]. While [48] speculated that insects could have obtained the ability to synthesize adenylate CKs via horizontal gene transfer or microbial symbiosis, the insect’s own *t*RNA-*ipt* pathway or potentially the same pathway in a microbial symbiont seem to be the most probable pathway for CK synthesis. In the green-island-inducing *P. blancardella*, where a microbial symbiont has been implicated as the source of the observed CKs, the symbiont is a strain of *Wolbachia* [54,55,93]. Interestingly, of the more than 44 *Wolbachi*a genomes sequenced thus far, no strain has yet been shown to have the adenylate IPT pathway, but they all possess the *t*RNA-*ipt* pathway.

If insects produce CKs via the *t*RNA-*ipt* pathway alone, in combination with microbial symbionts, or solely via microbial versions of this pathway, then the concentrations and composition of CKs reported in this survey are not consistent with the types and amounts of CKs produced by this pathway in plants. To date, plant-based studies suggest that the *t*RNA-*ipt* pathway results solely in the production of *c*Z forms of CKs [2,35]. Furthermore, the *t*RNA-*ipt* pathway in plants is argued not to be sufficiently productive to account for more than 40% of the CKs observed in plants [94], partly because of the low turnover rate of *t*RNA, and hence the limited source of *t*RNAs to release free CKs. However, recent work with the gall-inducing fungus *U. maydis* and other fungi, along with the pathogenic bacterium *M. tuberculosis,* suggests that in addition to *c*Z, both isopentenyladenine (iP) and methylthiolated cytokinins (METs) can also be produced via the *t*RNA pathway [6,11,27].

Most of the insects we examined, and those examined in other studies of gall- or green-island-inducing insect species, had high concentrations of *i*P and *t*Z as well as those of their RB precursors. The extraordinarily high concentrations and altered composition of CKs in insects suggest that the enzymes and substrates involved in CK synthesis and metabolism might differ from those in plants. All eukaryotes, except plants, appear to have iP associated with *t*RNA at adenine residue 37 [95] and this might account for the altered composition of CKs in insects relative to plants. In the moss *Physcomitrella patens*, which has several versions of IPT, knockout of IPT1 terminated prenylation of *t*RNA and led surprisingly to increased concentrations of iP and *t*Z forms of CKs [96]. This result led to the suggestion that a *t*RNA independent pathway contributes to CK production in *P. patens,* possibly involving prenylation of AMP, ADP, or ATP by one of the other IPT genes. However, in insects, only a single IPT gene has been reported, so if CK synthesis is *t*RNA independent in insects, it must involve some as yet undescribed non-canonical pathway. A broad analysis of the evolution of isopentenyltransferase genes grouped insect and other animal versions of the *t*RNA dimethylallytransferase genes in a clade wedged between a clade including slime molds and yeasts, and a clade containing higher plants, again supporting the possibility that animal forms of this gene may function differently than plant or bacterial forms [31]. Perhaps the *t*RNA-*ipt* enzyme in insects can also use other substrates such as rRNA, micro RNAs, *t*RNA fragments, or AMP, ADP, or ATP [97]. More extensive research on CK biosynthesis in insects is needed to better understand the production of such high levels of *t*Z and iP forms of CK in insects.

### 3.10. Role of Cytokinins in Gall Induction

Our results and those of [18] suggest that CKs in insects are not exclusively associated with gall induction; however, we are not questioning the role of CKs in gall induction. It has long been suggested that the combination of high concentrations of CKs and auxin (IAA) in gall-inducing insects either completely accounts for, or at least contributes to, the proliferation of plant tissues that constitutes the covering gall [15,16,41,46,48,98]. Therefore, information on the distribution of auxin in Insecta is a critical step toward determining how the joint distribution of CKs and auxin associates with the gall-inducing phenotype. Better understanding of the role of CKs in gall induction by insects and insect manipulation of their host plants via phytohormones will require additional data on the micro-distribution and concentration of auxin and other plant hormones within insects.

While we find the NT and RB precursors of CKs to be abundant and widespread in both gall- and non-gall-inducing insects, the more active FB forms are more common in gall-inducing insects in the Thysanoptera, Hymenoptera, and Diptera, and in non-gall-inducing insects in the Hemiptera, Coleoptera, and Lepidoptera (Tables S1–S4). The wide diversity in concentration and composition of CKs found in gall- and non-gall-inducing insects raises the question of what types and levels of CKs are necessary for successful gall induction. The values reported in this study are whole-body levels rather than estimates for the glands that may be involved in delivering CKs to the host-plant. After micro-dissections of adults, [46] measured CKs and auxin analytes of the accessory glands associated with the ovipositor in *Pontania* sp. (Hymenoptera: Tenthredinidae). They reported total CK concentration of 430,760 pmol/g fwt (predominantly *t*ZR) and IAA concentration of 154 pmol/g fwt in the accessory glands of adult females. In stark contrast to the values reported for the accessory glands, their measurements of the whole-body concentrations of CKs for larvae was only 5,963 pmol/g fwt, while for IAA it was 6,279 pmol/g fwt. As mentioned previously, in *Pontania* sp., the gall is induced by the female at oviposition and can reach 5 mm in diameter prior to larval hatching. Feeding by larvae results in further expansion of the gall. The CK-rich oviposition fluid is likely crucial to gall induction and the concentration of CKs in the larval salivary glands may be equally high. However, without gland- or tissue-specific measurements of phytohormone concentrations, and potentially concentrations in the salivary or oviposition fluids, it will be difficult to establish the phytohormone titer necessary for gall induction. For the gall-inducing beetle *R. pilosa* for which we found low whole-body concentrations of CKs (83 pmol/g fwt), the concentrations in oviposition fluids, which are entirely responsible for gall induction, could be substantially higher. After isolating ovipositional fluid from *R. pilosa* for their analyses of phenolic compounds, [99] found higher concentrations for at least some of these metabolites in the oviposition fluid than in extracts from whole females.

Production of phytohormones by gall-inducing insects is insufficient to account for gall induction, since we found non-gall-inducing insects to harbor a variety of CKs sometimes at high levels, and [100] also reported auxin from non-gall-inducing insect species. What then accounts for gall induction? While [101] suggests that secreted effector proteins are responsible for gall induction, this inference is based mainly on the claim that the Hessian fly genome reveals a large increase in the number of putative effector proteins. The claim that secreted effector proteins account for gall induction is not supported by any direct evidence. Furthermore, only a few effector proteins have been shown to be secreted extra-cellularly or linked with Hessian fly virulence/avirulence at present [102,103,104]. Furthermore, in the Hessian fly system, no covering gall is present, so whatever effectors may be found could only contribute to the generation of the nutritive layer. Nonetheless, it is possible that effector proteins [105], or other effectors in addition to phytohormones, contribute to gall induction. Further research will be necessary to either establish or rule out a contribution for proteinaceous or other non-protein effectors in gall induction.

On the other hand, gall-inducing insects differ from non-gall-inducing insects in that they are specialists in attacking “reactive sites” on plants [106,107,108,109]. Such reactive sites are meristematic regions of the plant that include undifferentiated or only partly differentiated stem cells that are capable of the cell division and tissue growth required to generate plant galls [108,109]. We hypothesize that interactions of secreted phytohormones with stem cells found in meristems give rise to proliferations of plant tissue that constitute covering galls. Primary and secondary (cambial) meristems are under the control of CKs and IAA, and cell division, differentiation, and dedifferentiation can result from gradients in their concentration [110,111]. Therefore, we contend that what separates gall- from non-gall-inducing insects is their specialization to attack meristems, rather than their ability to produce phytohormones, which our results suggest is widespread among phytophagous insects.

### 3.11. Role of Cytokinins in Manipulating Nutrient Allocation and Host-Plant Defenses

CKs detected in insects can be deployed against plants for at least two, possibly related benefits: altering nutrient availability, plant defenses, or both. CKs have been shown to lead to the formation and strengthening of mobilizing sinks to which photosynthates are translocated from other plant modules [1,60,112,113]. We report that CKs are present in every species of phytophagous insect we examined, not just gall-inducing and leaf-mining species. Insect-induced plant galls, insect-induced green islands, and the feeding sites of a group of mirid bugs are all sites into which the insects secrete exogenous CKs [15,16,18,19,44,46,47,49,50,52]. Among more sedentary insects, production and secretion of exogenous CKs likely leads to the formation of mobilizing sinks, resulting in the translocation of sugars and other compounds to the feeding site of the insect [18,58,59]. As mobilizing sinks, the plant module hosting the feeding site will provide adequate nutrition to the insect, and is unlikely to be abscised in spite of the damage caused by the insect.

In addition to their role in establishing sinks, CKs from insects may also alter plant defensive responses to insect feeding. Support for this hypothesis would be strengthened if CKs were found in saliva or salivary glands of these insect species, but exploring this detail will require a targeted research effort. When caterpillars feed on leaves, CK levels increase and the response of plant defense pathways is complicated, with some plant defensive pathways that are being regulated by CKs increasing and other indirect defense pathways not being affected [62]. However, the CK concentrations we report are much higher than those used by [62] (~9 pmol/g fwt), and how plants may respond to potentially much higher concentrations of CKs secreted by insects is as yet unknown. Several transcriptomic studies of gall systems report that gene expression in plant defense pathways are either unaffected or down-regulated in galls relative to un-galled plant tissues [19,114,115,116,117,118,119,120], while other studies make no mention of effects of galling on host defenses or report up-regulation of plant defensive pathways [121,122,123,124,125]. Given the complicated relationships among phytohormones [126], there is likely to be crosstalk between the CK signaling pathway and other plant defense pathways, so perhaps CKs modulate defense. It certainly is suggestive that a range of plant antagonists, including viruses, protists, bacteria, nematodes, and fungi, produce CKs apparently to facilitate plant invasion [60], so perhaps CKs in insects somehow function similarly. For auxins, which, like CKs, have been suspected to be involved in gall induction, it has been hypothesized that the initial ability of gall-inducing insects to produce indole-3-acetic acid (IAA) may have first evolved to subdue plant defense responses, then other selection pressures drove IAA production up even further [127,128]. Perhaps a similar scenario can be envisioned for CKs. Under this scenario, the initial ability of insects to produce CKs provided advantages to those individuals in the population because they were able to moderate plant defenses, more easily establish sinks, or both, before selection drove further increases in CKs to the levels we detected in this study.

The widespread occurrence of CKs in phytophagous insects suggests that the primary role of insect-derived CKs is acting as means for herbivorous insects to manipulate their host-plant species by modifying nutrient flux during herbivory to direct nutrients to the insect’s feeding site. We hypothesize that obvious phenotypic manipulations associated with CKs, such as the induction of galls or green islands, are secondary to manipulations of nutrient allocation and plant defense. Additionally, we speculate that insect-derived CKs may have a role in altering plant defensive responses.

## 4. Conclusions

Our results show that CKs are widespread and abundant in phytophagous insects suggesting that their injection into host plants during feeding or oviposition may be a common strategy to manipulate host plants. CKs are not unique to gall- or green-island-inducing insects alone. The widespread occurrence of CKs in phytophagous insects suggests that the primary roles of insect-derived CKs is to allow herbivorous insects to manipulate source-sink mechanisms of nutrient allocation in the host plant and to modify plant defenses typically deployed against insects. Furthermore, the higher concentrations and altered composition of CKs in insects in comparison to plants suggest that biosynthesis of CKs in insects may involve specific modifications of the *t*RNA-*ipt* pathway, which increases efficiency of CK production and diversifies the forms of CKs in comparison to the types believed to arise via the *t*RNA-*ipt* pathway in plants. Further understanding of the role of CKs in gall induction and insect manipulation of their host plants via phytohormones will require more data on the phylogenetic and micro-spatial distribution and concentrations of CKs and auxin within and among insects.

## Figures and Tables

**Table 1:** Tissue sampling for cytokinin profiling analysis.

Order (Family)	Gall Inducer	Non-Gall Inducer	Life Stage	Host Plant	Locality	Site-Collector
**Thysanoptera**						
(Phlaeothripidae)	*Klambothrips myopori*		Adult	*Myoporum laetum*	Pacifica, CA	Field-EFC
(Thripidae)		*Frankliniella occidentalis*	Adult	*Rosa* sp.	Half Moon Bay, CA	Greenhouse-EFC
Hemiptera						
(Aphididae)	*Tamalia coweni*		Adult	*Arctostaphylos viscida*	Chico, CA	Field-DGM & EFC
		*Tamalia inquilinus*	Adult + Nymph	*A. viscida*	Chico, CA	Field-EFC
		*Myzus persicae*	Adult + Nymph	*Salix lasiolepis*	San Francisco, CA	Field-EFC
**Coleoptera**						
(Curculionidae)	*Rhinusa pilosa*		Adult & Larvae	*Linaria vulgaris*	Lethbridge, CAN	Lab Colonies-RDF
		*Mecinus janthinus*	Adult & Larvae	*L. vulgaris*	Lethbridge, CAN	Lab & Field colonies-RDF
		*Mecinus janthiniformis*	Adult & Larvae	*Linaria dalmatica*	Fort Macleod, CAN	Field-RDF
**Hymenoptera**						
(Tenthredinoidea)	*Pontania pacifica*		Larvae	*S. lasiolepis*	Pacifica, CA	Field-EFC
		*Cimbex americana*	Larvae	*S. lasiolepis*	Pacifica, CA	Field-EFC
		*Nematus iridescens*	Larvae	*Populus angustifolia* x *P. fremontii*	Flagstaff, AZ	Field-PWP
**Lepidoptera**						
(Gelechiidae)	*Gnorimoschema gallaesolidaginis*		Larvae	*Solidago altissima*	University Park, PA	Field-JFT
		*Dichomeris* sp.	Larvae	*S. altissima*	University Park, PA	Field-JFT
**Diptera**						
(Tephritidae)	*Eurosta solidaginis*		Adult & Larvae	*S. altissima*	Northfield, MN	Field-EFC
		*Bactrocera oleae*	Adult & Larvae	*Olea europea*	Santa Rosa, CA	Field-EFC
(Cecidomyiidae)	*Mayetiola destructor*		Larvae	*Triticum aestivum*	Manhattan, KS	Greenhouse-MSC
	*Rhopalomyia californica*		Larvae	*Baccharis pilularis*	San Francisco, CA	Field-EFC

In cases where two life stages were collected: & indicates both stages were analysed separately, + indicates stages were pooled. Collectors are indicated by abbreviated names: Rosemarie De Clerck-Floate (RDF), Edward F. Connor (EFC), Ming-Shun Chen (MSC), John F. Tooker (JFT), Peter W. Price (PWP), Donald G. Miller III (DGM).

**Table 2:** Names and abbreviations of endogenous cytokinins (CKs) and labeled CK standards, scanned for by liquid chromatography-positive electrospray ionization tandem mass spectrometry (HPLC-(ESI+)-MS/MS). Deuterated internal standards purchased from OlChemim Ltd. (Olomouc, Czech Republic) were used for the analysis.

Isoprenoid Cytokinins		Labeled CK Internal Standard
**Nucleotides (NTs)**		
1.	*Trans-*zeatin riboside-5′-monophosphate (*t*ZNT)	^2^H_5_[9RMP]Z
2.	*Cis-*zeatin riboside-5′-monophosphate (*c*ZNT)	
3.	Dihydrozeatin riboside -5′-monophosphate (DHZNT)	^2^H_3_[9RMP]DHZ
4.	Isopentyladenosine-5′monophosphate (iPNT)	^2^H_6_[9RMP]iP
**Ribosides (RBs)**		
5.	*Trans-*zeatin riboside (*t*ZR)	^2^H_5_[9R]Z
6.	*Cis-*zeatin riboside (*c*ZR)	
7.	Dihydrozeatin riboside (DHZR)	^2^H_3_[9R]DHZ
8.	Isopentyladenosine (iPR)	^2^H_6_[9R]iP
**Free bases (FBs)**		
9.	*Trans-*zeatin (*t*Z)	^2^H_3_DHZ
10.	*Cis-*zeatin (*c*Z)	
11.	Dihydrozeatin (DHZ)	
12.	Isopentyladenine (iP)	^2^H_6_iP
**Glucosides (GLUCs)**		
13.	*Trans*-zeatin-O-glucoside (*t*ZOG)	^2^H_5_ZOG
14.	*Cis*-zeatin-O-glucoside (*c*ZOG)	
15.	Dihydrozeatin-O-glucoside (DHZOG)	^2^H_7_DHZOG
16.	*Trans*-zeatin-O-glucoside riboside (*t*ZROG)	^2^H_5_ZROG
17.	*Cis*-zeatin-O-glucoside riboside *c*ZROG	
18.	Dihydrozeatin-O-glucoside riboside (DHZROG)	^2^H_7_DHZROG
19.	*Trans*-zeatin-9-glucoside (*t*Z9G)	^2^H_5_Z9G
20.	*Cis*-zeatin-9-glucoside (*c*Z9G)	
21.	Dihydrozeatin-9-glucoside (DHZ9G)	^2^H_3_DHZ9G
22.	Isopentenyladenine-7-glucoside (iP7G)	^2^H_5_iP7G
23.	Isopentenyladenine-9-glucoside (iP9G)	
**Methylthiols (METs)**		
24.	2-Methylthio-*trans*-zeatin (2MeSZ)	^2^H_5_MeSZ
25.	2-Methylthio-*trans*-zeatin riboside (2MeSZR)	^2^H_5_MeSZR
26.	2-Methylthio-isopentyladenine (2MeSiP)	^2^H_6_MeSiP
27.	2-Methylthio-isopentyladenosine (2MeSiPA)	^2^H_6_MeSiPR
**Aromatic Cytokinins**		
28.	Benzylaminopurine (BA)	^2^H_7_BA
29.	Benzylaminopurine riboside (BAR)	^2^H_7_BAR

**Table 3:** Concentrations of CK forms (FB, RB, NT) for trans-zeatin (*t*Z), cis-zeatin (*c*Z), and isopentyladenine (iP) expressed as means ± standard error (SE) in pmol/g fwt of whole-body mass for gall-inducing (GI) and non-gall-inducing (NGI) insect species; empty cells indicate analytes not detected. In cases where two life stages were collected, + indicates stages were pooled. D3 indicates day 3 larvae for *M. destructor*.

Insect Order & Species	*t*Z +*t*ZR + *t*ZNT Mean ± SE	*c*Z + *c*ZR + *c*ZNT Mean ± SE	iP + iPR + iPNT Mean ± SE	Total CKs (all forms) Mean ± SE
Thysanoptera				
***F. occidentalis* (NGI) Adult**	43 ± 10	23 ± 7	3730 ± 281	4876 ± 376
***K.myopori* (GI) Adult**	869 ± 377	28 ± 6	2799 ± 265	5548 ± 945
Hemiptera				
***T. coweni* (GI) Adult**	191 ± 68	4 ± 3	2323 ± 196	3369 ± 220
***T. inquilinus* (NGI) Adult + Nymph**	120 ± 35	11 ± 4	2030 ± 96	3068 ± 169
***M. persicae* (NGI) Adult + Nymph**	1290 ± 64	1295 ± 33	2705 ± 109	7436 ± 177
Coleoptera				
***R. pilosa* (GI) Larvae**				2439 ± 1172
*M. janthinus* (NGI) Larvae	12,910 ± 1584	12,550 ± 2464	3865 ± 3283	30,928 ± 499
***M. janthiniformis* (NGI) Larvae**	50,287 ± 10,134	49,268 ± 9383	11,765 ± 4023	111,630 ± 21,383
***R. pilosa* (GI) Adult**	2 ± 0.7	2 ± 0.7	76 ± 8	83 ± 8
***M. janthinus* (NGI) Adult**				1961 ± 352
***M. janthiniformis* (NGI) Adult**	16 ± 16		6 ± 6	13,184 ± 6687
Hymenoptera				
***P. pacifica* (GI) Larvae**	676 ± 314		109 ± 31	848 ± 330
***C. americana* (NGI) Larvae**		2 ± 0.4	25 ± 6	29 ± 6
***N. iridescens* (NGI) Larvae**	14 ± 2	44 ± 2	154 ± 35	463 ± 74
Lepidoptera				
***G. gallaesolidaginis* (GI) Larvae**	96 ± 53	104 ± 67	252 ± 78	526 ± 167
***Dichomeris* sp. (NGI) Larvae**	2 ± 1.5	54 ± 17	849 ± 130	1424 ± 302
Diptera				
***E. solidaginis* (GI) Larvae**	154,251 ± 40,883		54,646 ± 4191	318,405 ± 47,781
***B. oleae* (NGI) Larvae**			71 ± 11	138 ± 11
***E. solidaginis* (GI) Adult**	12 ± 1		695 ± 174	769 ± 191
***B. oleae* (NGI) Adult**			475 ± 354	989 ± 473
***M. destructor* (GI) Larvae D3**	181 ± 28	754 ± 57	151 ± 20	2600 ± 72
***R. californica* (GI) Larvae**	486 ± 163	631 ± 252		1118 ± 401

## Supplementary Materials

The following are available online at https://www.mdpi.com/2223-7747/9/2/208/s1. Table S1: Cytokinin (CK) concentrations [pmol/g fwt of whole-body mass] in insects of Thysanoptera and Hemiptera. Table S2. Cytokinin (CK) concentrations [pmol/g fwt of whole-body mass] in insects of Coleoptera. Table S3. Cytokinin (CK) concentrations [pmol/g fwt of whole-body mass] in insects of Lepidoptera and Hymenoptera. Table S4. Cytokinin (CK) concentrations [pmol/g fwt of whole-body mass] in insects of Diptera.
